# Measuring turfgrass canopy interception and throughfall using co-located pluviometers

**DOI:** 10.1371/journal.pone.0271236

**Published:** 2022-09-02

**Authors:** Don Wesley Dyer, Andres Patrignani, Dale Bremer

**Affiliations:** 1 Department of Horticulture and Natural Resources, Kansas State University, Manhattan, Kansas, United States of America; 2 Department of Agronomy, Kansas State University, Manhattan, Kansas, United States of America; Ningbo University, CHINA

## Abstract

Turfgrass management relies on frequent watering events from natural precipitation or irrigation. However, most irrigation scheduling strategies in turfgrass ignore the magnitude of canopy interception. Interception is the process by which precipitation or irrigation water is intercepted by and evaporated from plant canopies or plant residue. The objective of this study was to quantify the magnitude of precipitation interception and throughfall in ‘Meyer’ zoysiagrass (Zoysia japonica L.) and ‘007’ creeping bentgrass (*Agrostis stolonifera* L.). We used a new method consisting of co-located pluviometers with and without circular turfgrass patches to measure interception and throughfall. The resulting dataset includes 15 storms and 25 individual rainfall events ranging in precipitation totals from 0.3 mm to 42.4 mm throughout the research study. Throughfall amount resulted in a strong (*r* = 0.98) positive linear relationship with precipitation totals. On average, zoysiagrass and creeping bentgrass canopies intercepted a minimum of 4.4 mm before throughfall occurred. This indicates that, on average, no precipitation reaches the soil surface for precipitation events <4.4 mm. After the point of throughfall, 16% of each additional millimeter of precipitation or irrigation is lost due to interception. Nearly, 45% of the area of the contiguous U.S. could result in >50% of the annual precipitation being intercepted by canopies of zoysiagrass and bentgrass. This study provides detailed insights to understanding the interception dynamics in turfgrass and highlights the inefficient nature of small precipitation and irrigation events in turfgrass systems.

## Introduction

In the United States, there are more than 12.5 million hectares of irrigated turfgrass [1]. Golf courses alone account for 600,000 hectares of turf that use approximately use 2.2 km^3^ of water per year [2]. Turfgrass plays an important role in recreational spaces, sport fields, and landscaping both for aesthetic purposes and to prevent soil erosion. Inevitably, the shallow (i.e., <30 cm) root system usually makes turfgrass vulnerable to soil water deficits, thus, irrigation is typically an integral component of turfgrass management. To better guide in-season irrigation decisions, such as irrigation amounts and frequencies, irrigation scheduling in turfgrass requires accurate knowledge of the components of the soil water balance [3]. While traditional irrigation scheduling involves fixed watering amounts and frequencies, improved irrigation decisions aimed at conserving water resources typically integrate meteorological and soil moisture information to assess the ability of turfgrass to cope with the atmospheric demand given the available rootzone soil water capacity [2]. However, a component of the soil water balance that is often neglected in irrigation prescriptions is the magnitude of both natural precipitation and irrigation interception by the turfgrass canopy, which can reduce the amount of precipitation and irrigation water reaching the rootzone.

Interception can be defined as precipitation or irrigation water that is prevented from reaching the soil surface by plant canopies or surface litter. Intercepted droplets can remain on the surface of leaves, stems, and litter, and then evaporate into the atmosphere during and after precipitation events [4, 5]. As a result, interception is often considered a loss in the soil water balance [6, 7]. In formal terms, interception can be defined as [8]:

I=P‐TF [Eq 1]

where *I* represents canopy and litter interception (mm), *P* is precipitation (mm), and *TF* is throughfall (mm). Throughfall is defined as the amount of precipitation or irrigation water that passes through the canopy. For clarity, in this study we limit the use of the term “precipitation” to denote liquid precipitation. In trees and shrubs there is often an additional term for stemflow, which is the water that flows down along branches and the main stem. Unlike trees and shrubs, turfgrass systems are uniquely characterized by a dense plant canopy that can propagate by stolons and/or rhizomes. Thus, mature turfgrass canopies typically develop a thatch layer of intermingled dead and living material between the actively growing canopy and the soil surface that can restrict and hold precipitation and irrigation water [9, 10]. For instance, previous studies suggested that creeping bentgrass (*Agrostis stolonifera* L.) could retain an amount of water equivalent to 50% of the thickness of the thatch layer [11]. Thus, in this study we use the term throughfall to denote the additive combination of both throughfall and stemflow.

Previous studies have extensively investigated canopy interception in land covers other than turfgrass. For example, a forage sorghum (*Sorghum bicolor* L. Moench) canopy in a humid subtropical climate in Oklahoma, US intercepted 27–45% of the growing season rainfall [12]. In a tallgrass prairie dominated by big bluestem (*Andropogon gerardii*), little bluestem (*Schizachyrium scoparium*), and Indiangrass (*Sorghastrum nutans*) in the Flint Hills region in Kansas, US, mean canopy interception throughout a two-year study accounted for 38% of annual rainfall [13]. A study in a coastal redwood (*Sequoia sempervirens*) and Douglas-fir (*Pseudotsuga menziesii*) forest in northwest California, US revealed that about 22% of the annual precipitation is evaporated from the foliage and stems [14]. Therefore, canopy interception can play an important role in the fraction of annual precipitation that reaches the soil surface. Across most interception studies, the amount of canopy interception is related to plant canopy characteristics such as leaf area index and biomass, and meteorological factors such as rainfall amount, duration, intensity, and atmospheric evaporative demand. While considerable research has been conducted to show the impact of canopy interception in other land covers, to our knowledge no prior study has quantified the magnitude of canopy interception and throughfall in turfgrass. Our study introduces a simple method for measuring turfgrass canopy interception and throughfall at high temporal resolution. The new method can be adapted to multiple turfgrass species and takes into account natural rainfall events. Unfolding this unknown component of the soil water balance could be a key element for a more efficient use of water turfgrass systems. The objective of this study was to quantify the magnitude of precipitation interception and the timing of canopy throughfall in ‘Meyer’ zoysiagrass (Z*oysia japonica* Steud.) and ‘007’ creeping bentgrass using a new method consisting of co-located pluviometers.

## Materials and methods

The study was conducted at the Rocky Ford Turfgrass Research Center near Manhattan, Kansas (39°13’59.628” N, 96°34’30.612” W, 315 m a.s.l.) during September and October 2019 and from March to June 2020. The study site is characterized by an average annual temperature of 13.4°C and an average annual rainfall of 895 mm that is concentrated during the late spring and summer months. The site belongs to the Dfa Köppen-Geiger climate classification, which is characterized by humid continental hot summers with year-round precipitation [15]. The selected grasses represent two of the most widely used turfgrass in the United States. Zoysiagrass is a turfgrass widely used in transitional and warm climatic regions and often requires minimal maintenance and low inputs [16], while bentgrass is well-adapted to cool and humid climates [17].

**Fig 1:**
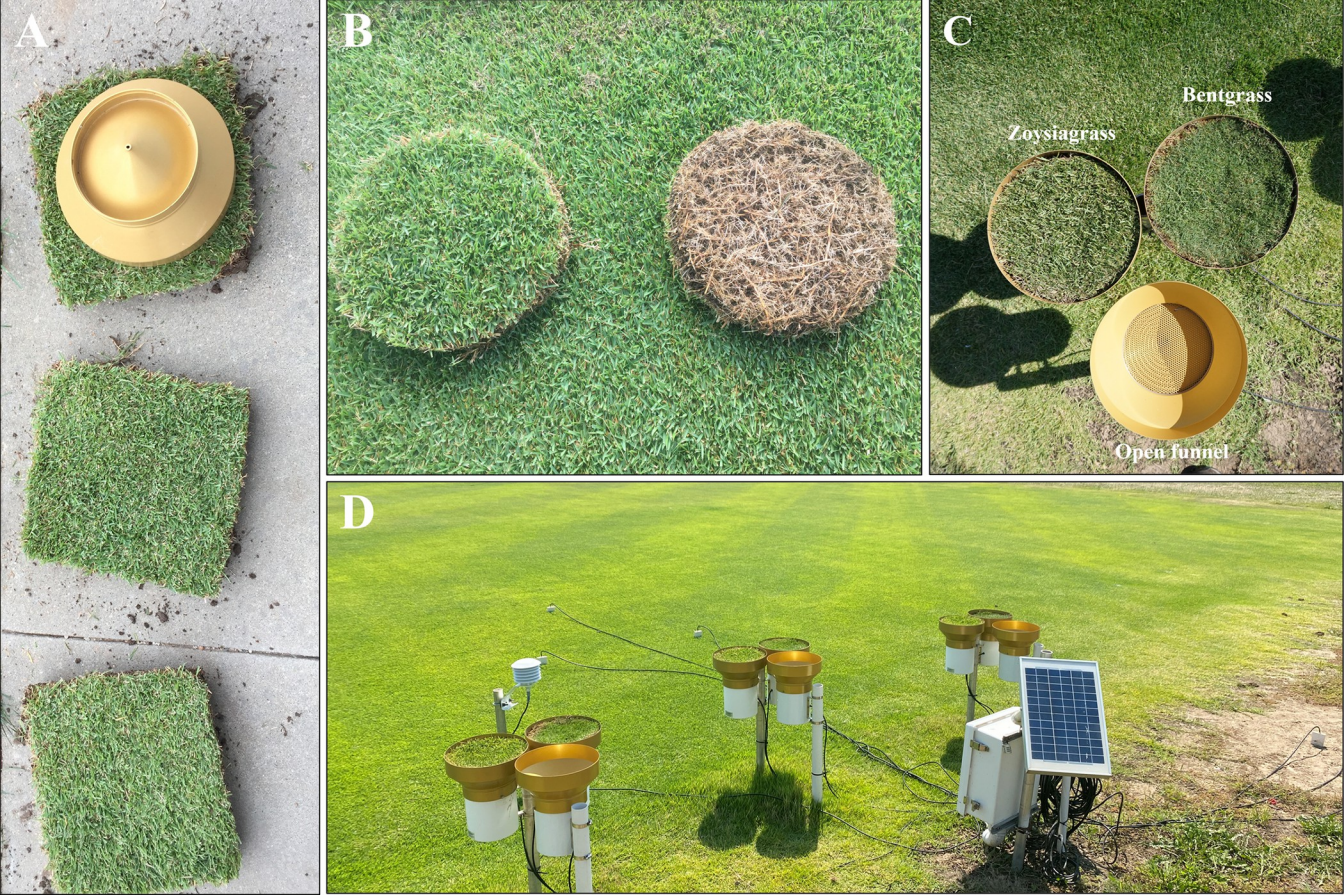
Figure illustrating A) the process of delineating and cutting the turfgrass patch using the pluviometer collector as template to ensure close fit, B) the top and bottom of a turfgrass patch after removing the soil attached to the bottom of the thatch layer and ready to be inserted into the pluviometer collector, C) a top view example of the pluviometers with and without the turfgrass patches of zoysiagrass and creeping bentgrass before the occurrence of a precipitation event, and D) the replicated field set up with showing three sets of three co-located pluviometers, a sensor for measuring air temperature and relative humidity, soil moisture sensors deployed in bare soil and below the surrounding zoysiagrass canopy, and associated logging hardware.

Throughfall and precipitation interception were measured simultaneously using a new approach consisting of co-located pluviometers with and without circular turfgrass patches inserted into the pluviometer funnel. A few hours before each storm, a new set of turfgrass patches of zoysiagrass and creeping bentgrass were cut, cleaned from debris, and then placed inside the pluviometer funnel that had an opening with a diameter of 24.5 cm (Fig 1A–1C). The turfgrass patch encompassed the canopy leaves and the thatch layer, all roots below the thatch layer were removed. The turfgrass heights were maintained at 16 mm in the zoysiagrass and 12.7 mm in the bentgrass to mimic typical golf course fairway heights for each turfgrass. The thickness of the patch was determined by placing the turfgrass patch between a benchtop and a rigid disk that had the same area of the patch with a mass of 1 kg on top of it. This procedure allowed us to consistently measure the thickness of all patches. At the end of each storm, canopy storage capacity was determined by completely submerging each patch in a bucket with water for 5 minutes and then allowed to drip for 1 minute on a rack before recording the mass representing the maximum storage capacity of the patch. Then, patches were oven-dried at 105°C for 48 hours to determine the dry mass of the patch.

For this experiment we used a total of nine tipping-bucket pluviometers grouped in triplets to ensure replication of the experiment (Fig 1D). Each triplet had one open pluviometer (model TE525MM, Texas Electronics Inc., resolution of 0.1 mm per tip), one pluviometer covered with a patch of zoysiagrass, and one pluviometer covered with a patch of bentgrass (Fig 1C and 1D). Each pluviometer triplet was mounted on a pole at 0.75 m above the ground. This height was an arbitrary, but practical choice to ensure turfgrass patches were correctly placed before a storm. All pluviometers were calibrated following the manufacturer’s recommendation using a Mariotte’s bottle dispensing water at a rate of 473 ml of water in 45 minutes. All sensors met the factory requirement of 100 ± 3 tips for this amount of water. In addition to precipitation, relative humidity and air temperature were monitored using a sensor (model CS215, Campbell Scientific) mounted at a height of 1.2 m. For storms in 2020, changes in soil water storage were monitored using soil moisture sensors (model CS655, Campbell Scientific) installed vertically (0–12 cm depth) in adjacent areas of bare soil and zoysiagrass. A datalogger (model CR1000, Campbell Scientific) was programmed to record all variables at one-minute intervals, which allowed for detailed information of precipitation and throughfall measurements.

In the data analysis stage, we used a minimum inter-event time (MIT) criterion of one hour without measurable rainfall in the open pluviometers to identify individual rainfall events within a given storm [18, 19]. This criterion was selected to differentiate intra-storm precipitation events while still capturing low amount (~0.1 mm, resolution of the pluviometer) precipitation events as a single event. The Python programming language was utilized to read and process the 1-minute data and identify the individual precipitation events using the selected MIT. Time series for each gauge with the same patch treatment were averaged.

## Results and discussion

During the study period we captured a total of 15 storms and 25 individual precipitation events. Canopy throughfall and interception were measured for all storms in zoysiagrass and for ten out of the 15 storms in bentgrass (Table 1), using a total of 75 different turfgrass patches. Storm precipitation totals ranged from 0.4 mm to 42.4 mm (Table 1), values that are similar to the 1^st^ percentile (i.e., 0.25 mm d^-1^) and 99^th^ percentile (i.e., 57 mm d^-1^) estimated from daily precipitation records for the 2010–2020 period for the Manhattan station of the Kansas Mesonet [20], which is located 2.7 km from the experimental site. Among the three open pluviometers, the average difference between the lowest and highest recorded precipitation total for all precipitation events was typically 0.5 mm and the coefficient of variation was 1.8%. The storm with the largest number of individual precipitation events occurred on 1 October 2019 (storm 3), totaling four events. The longest duration for a single precipitation event lasted 16.1 hours (storm 5a) and the shortest precipitation event lasted only 23 minutes (storm 12a). The highest maximum rainfall intensity of 97 mm h^-1^ was recorded at the minute level for a storm on 22 September 2019 (storm 1a). Thus, our study covered a wide range of precipitation durations, amounts, and intensities typical for the central U.S. Great Plains [21].

**Table 1:** Table showing the duration, gross precipitation (*P*_*g*_), precipitation maximum intensity (*P*_*imax*_), throughfall (*TF*), interception until the point of throughfall (*I*_*tf*_), and canopy interception (*I*) for each individual precipitation event across 15 storms for turfgrass patches of ‘Meyer’ zoysiagrass and creeping bentgrass. Canopy interception values in parenthesis represent the percentage of *P*_*g*_. Values for each precipitation event are the average of three pluviometers.

				Zoysiagrass			Bentgrass		
Storm-Event	Duration	P_g_	P_imax_	TF	I_tf_	I	TF	I_tf_	I
	minutes	mm	mm hr^-1^	mm	mm	mm	mm	mm	mm
1-a	86	40.3	97	32.9	6.4	7.4 (18)			
2-a	102	4.3	28	0.1	3.4	4.2 (98)			
2-b	66	1.7	22	0.7	1.1	1.1 (62)			
3-a	40	14.7	88	8.5	2.4	6.2 (42)			
3-b	211	3	18	0.8	1.7	2.2 (74)			
3-c	148	5.3	22	4.6	0.3	0.7 (14)			
3-d	74	5.4	60	5	0.3	0.4 (7)			
4-a	320	12	10	8.5	2.7	3.6 (30)			
4-b	37	0.3	4	0	―	0.3 (100)			
5-a	968	42.4	14	29.8	5.7	12.6 (30)			
6-a	178	2.3	6	0	―	2.3 (100)	0	―	2.3 (100)
7-a	390	8.7	12	5.3	3.3	3.4 (39)	4.5	3.4	4.2 (49)
7-b	347	1.7	4	1.4	0.3	0.3 (19)	1.1	0.5	0.6 (35)
8-a	38	0.5	4	0	―	0.5 (100)	0	―	0.5 (100)
9-a	24	0.3	2	0	―	0.3 (100)	0	―	0.3 (100)
9-b	265	1.3	10	0	―	1.3 (100)	0	―	1.3 (100)
10-a	120	3.9	8	0	―	3.9 (100)	0	―	3.9 (100)
10-b	363	6.1	18	4.3	1.9	1.8 (29)	3.3	2.2	2.9 (47)
10-c	305	1.4	6	0.9	0.1	0.5 (37)	0.8	0.2	0.7 (47)
11-a	32	1.2	6	0	―	1.2 (100)	0	―	1.2 (100)
11-b	27	0.1	2	0	―	0.1 (100)	0	―	0.1 (100)
12-a	23	0.4	4	0	―	0.4 (100)	0	―	0.4 (100)
13-a	241	25.8	72	19.9	5.4	5.9 (23)	18.1	7.1	7.7 (30)
14-a	141	13.2	62	8.6	3.5	4.6 (35)	8.2	4	5 (38)
15-a	360	17.3	44	9.8	5.3	7.4 (43)	9.1	4.3	8.2 (48)
Total	4906	213.6		141.1		72.6			39.3

**Fig 2:**
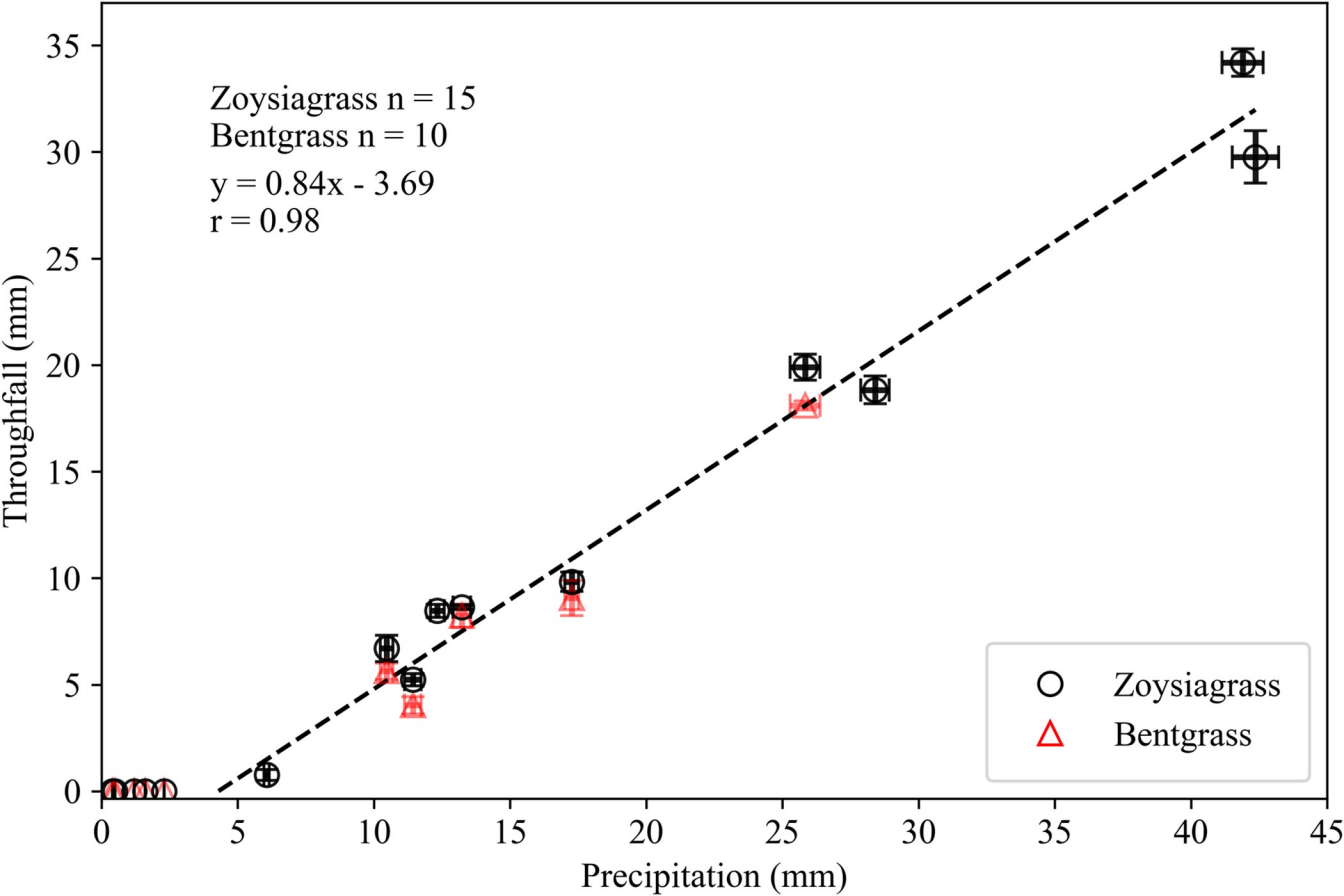
Relationship between precipitation and throughfall amount of patches of zoysiagrass and creeping bentgrass for all 15 natural storms. The *x*-intercept of 4.4 mm (95% CI [3.6, 5.3]) represents the minimum canopy interception before throughfall begins. The linear fitting exercise was done only using storm events that had throughfall >0 mm. Error bars represent the standard deviation of throughfall and precipitation. For some markers error bars are masked by the marker size.

Considering the total precipitation for all storms measured for each turfgrass, canopy interception losses accounted for 34% (73 out of 214 mm) in zoysiagrass and 47% (39 out of 84 mm) in bentgrass (Table 1). The relationship between gross precipitation and throughfall amount resulted in a strong positive linear correlation (*r* = 0.98), with an *x*-intercept of 4.4 mm (95% CI [3.6, 5.3]), and a slope of 0.84 (*P*<0.001) (Fig 2). In this context, the *x*-intercept represents the cumulative precipitation at the time throughfall (*I_tf_*). The slope of this relationship represents the precipitation losses due to interception after the point of throughfall. Thus, for zoysiagrass and bentgrass, only 84% of each additional millimeter of precipitation after the point of throughfall reaches the soil surface (Fig 2). Precipitation was completely intercepted by the turfgrass patches in five out of the 15 storms (Table 1). This is significant because the long-term median daily precipitation total for the study region is only 2.8 mm d^-1^ and only about 43% of the daily precipitation events at the study site are >4.4 mm. The interception at the point of throughfall found in this study for zoysiagrass and bentgrass was about 4 times larger than the *I_tf_* of 1.61 mm found for a Spruce (*Picea crassifolia* Kom.) forest in the semiarid mountain regions of China using 60 throughfall collectors [22]. Our results are also slightly higher than the reported *I_tf_* values of 3.9 mm for a mature tallgrass prairie grasses and 3.4 mm for a close stand of Redcedar (*Juniperus virginiana* L.) trees in central Oklahoma [23]. Similarly, a recent study in this region estimated that the interception of natural grassland vegetation typically results in 7.6 mm [24]. However, the study by Parker and Patrignani [24] also included in the interception estimate the soil water storage of the first ~2 cm of the soil profile, further indicating that our value of 4.4 mm is reasonable for this region when solely considering the turfgrass canopy and thatch layer. The slope of the precipitation-throughfall relationship found in our study also agrees well with previous studies in other land covers. For instance, the relationship between gross precipitation and throughfall in a matorral community in northeastern Mexico resulted in *r* = 0.99 and a slope of 85% [25].

The *x*-intercept and the slope of the relationship between gross precipitation and throughfall are of practical relevance to scientists and practitioners alike. Based on the findings reported in Fig 2, the daily interception amount could be approximated as follows:

$$I=P\;\mathrm{for}\;P\leq I_{tf}$$ [Eq 2]

$$I=I_{tf}+0.16(P-I_{tf})\;\mathrm{for}\;P>I_{tf}$$ [Eq 3]

where *I_tf_* is the cumulative precipitation at the time throughfall begins and 0.16 (i.e., 1−0.84) is the interception loss after throughfall (Fig 2). This relationship should provide a good first-order approximation for estimating the interception loss in zoysiagrass and creeping bentgrass mowed at standard heights for precipitation events up to ~45 mm d^-1^. Further research is required to estimate whether the *I_tf_* and slope found in this study can be used to estimate interception in other turfgrass canopies and other regions.

**Fig 3:**
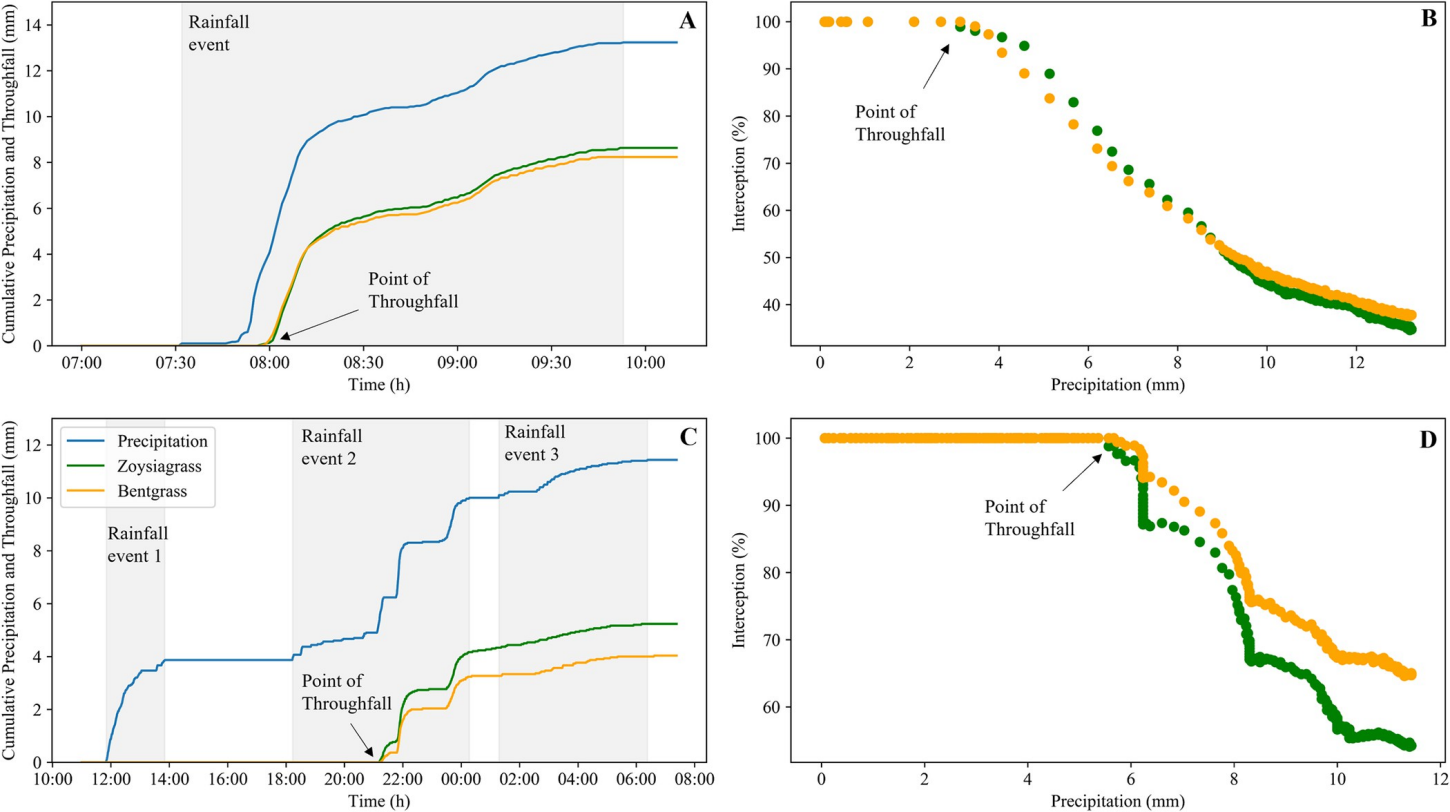
Comparison of cumulative precipitation, cumulative throughfall, and canopy interception for patches of zoysiagrass and creeping bentgrass during a storm with a single rainfall event (A and B, storm 14 in Table 1) and a storm with three rainfall events of variable lengths (C and D, storm 10 in Table 1). Both storms resulted in similar precipitation amount but had a different number of intra-storm precipitation events. The point of throughfall denoted by arrows.

To illustrate the impact of precipitation pulses and antecedent wetting events on canopy interception and throughfall, we compared two storms that resulted in similar precipitation amount, but that had contrasting duration and number of intra-storm precipitation events (Fig 3). The first storm (storm 14 in Table 1) occurred on 22 May 2020 and consisted of only one precipitation event totaling 13.2 mm over the period of 141 minutes (Fig 3A). During storm 14, the point of throughfall occurred in the zoysiagrass canopy after 3.5 mm and in bentgrass canopy after 4.0 mm of precipitation. The total interception amount for zoysiagrass was 4.6 mm and for bentgrass was 5 mm (Table 1, Fig 3B). The zoysiagrass canopy intercepted 35% and the bentgrass canopy intercepted 38% of the precipitation in storm 14. On the other hand, the second storm (storm 10 in Table 1) that occurred on 16 April 2020 had three intra-storm precipitation events and lasted a total of 18.5 hours (Fig 3C). In this storm, the first precipitation event with an amount of 3.9 mm was completely intercepted by both turfgrass patches. Throughfall eventually occurred during the second precipitation event, when the cumulative precipitation reached 5.8 mm in the zoysiagrass canopy and 6.1 mm in the bentgrass canopy. The first, second, and third precipitation events within the zoysiagrass canopy intercepted 100%, 29% and 37% of the precipitation and the bentgrass intercepted 100%, 47%, and 47% for each event during the storm, respectively. This decreasing interception percentage illustrates that the canopy interception capacity is highest at the start of a rainfall event and decreases with precipitation [26]. However, during rainless break periods water stored in the canopy can be lost to evaporation allowing the plant canopy to partially dry and restore some of its water storage capacity [27]. Considering the precipitation total for in storm 10, the zoysiagrass canopy intercepted 54% and the bentgrass canopy intercepted 65% of the precipitation (Fig 3B and 3D).

Detailed inspection of individual precipitation events using time series similar to those in Fig 3 revealed that canopy interception in turfgrass canopies had three well-defined stages. The first stage was characterized by complete precipitation interception by the canopy. During the first stage, droplets from precipitation and splashing can remain on top of leaves, stems, and litter, be evaporated, or be absorbed by plant tissue and organic material. The magnitude of each of these processes is likely dictated by the nature of the canopy and thatch layer, precipitation intensity, and the atmospheric demand during the precipitation event. The second stage was characterized by both throughfall and canopy interception, although the evaporation rate may be minor compared to throughfall amount due to the typically low (~0.1 kPa) vapor pressure deficit during rainfall events in this region [24]. The second stage exhibited a well-defined starting point (i.e., the point of throughfall, *I*_*tf*_) at which the canopy can no longer intercept all the precipitation, and therefore, additional water droplets move through the canopy and the thatch layer. The third stage consisted of the drying of the canopy after the precipitation has ceased, which includes some dripping and evaporation. Fig 3A and 3C show the timing of the third stage either at the end of a storm with a single rainfall event (Fig 3A) or during rainless periods in storms with multiple precipitation events (Fig 3C), which appear in a recurring cycle (Fig 3D).

The interception stages identified in our study are similar to those identified in a prior study investigating canopy interception of forest canopies [28], which were defined as: 1) a wetting phase as rainfall reaches the plant canopy, 2) a saturation phase as the plant canopy reaches its maximum water storage capacity, and 3) a drying phase after precipitation has ceased, in which the intercepted precipitation by the canopy evaporates from the external surface of the leaves and stems. Some of the main differences between the stages identified by Gash [28] and our study are evident in the second stage, in which the cumulative precipitation at the time of throughfall was nearly half of the measured interception storage capacity (*S*) using the submersion method. For instance, the zoysiagrass patches averaged *S* = 8.9 mm (SD = 1.3) and the bentgrass patches averaged *S* = 9.1 mm (SD = 1.4) (Table 2). The average interception at the point of throughfall was ~44% of the storage capacity for the zoysiagrass and ~50% for the bentgrass, thus illustrating throughfall occurs much earlier than the saturation point of the turfgrass canopy. Our findings indicate that at the point of throughfall, the amount of water held in the canopy does not necessarily match the storage capacity of the patch. The assumption of a “saturation phase” proposed by Gash [28] does not seem to apply in turfgrass.

**Table 2:** Storm, storage capacity, dry biomass, thatch layer for turfgrass patches of zoysiagrass and bentgrass. Value between parenthesis represent the standard error of the mean.

Storm	Storage Capacity	Dry Biomass	Patch Layer Thickness
	mm	g	mm
Zoysiagrass			
1	10.1 (0.3)	444 (12.3)	42 (0.5)
2	9.8 (0.4)	430 (9.1)	46 (0.4)
3	9.4 (0.4)	479 (18.9)	47 (0.4)
4	9.7 (0.2)	483 (3.2)	47 (0.5)
5	9.5 (0.6)	447 (20.8)	42 (0.4)
6	9.9 (0.4)	462 (22.5)	48 (0.3)
7	9.6 (0.7)	424 (8.5)	48 (0.7)
8	8.0 (0.5)	356 (51.6)	43 (0.2)
9	8.2 (0.7)	391(67.2)	46 (0.5)
10	9.6 (0.3)	402 (45.6)	46 (0.4)
11	6.1 (0.4)	325 (24.8)	45 (0.3)
12	7.7 (0.6)	485 (26.9)	47 (0.3)
13	7.1 (0.3)	332 (44.9)	46 (0.3)
14	11.1 (0.3)	361 (4.5)	45 (0.3)
15	9.4 (0.3)	578 (25.7)	47(0.2)
Bentgrass			
6	8.7 (0.9)	314 (13)	28 (0.4)
7	8.6 (0.2)	196 (35)	24 (0.4)
8	7.5 (0.4)	204 (23)	24 (0.7)
9	8.8 (2.6)	161 (19)	23 (0.5)
10	10.0 (0.7)	150 (19)	24 (0.4)
11	8.9 (0.5)	164 (29)	24 (0.4)
12	11.4 (1.4)	249 (56)	23 (0.4)
13	7.0 (2.8)	293 (75)	24(0.4)
14	10.9 (0.3)	337 (39)	26 (0.3)
15	9.4 (0.7)	316 (44)	24 (0.4)

The method used in this experiment to quantify canopy interception and throughfall does not allow for measurements of the evaporation rate from the turfgrass canopy during the third stage after precipitation has ceased. A previous study aimed at measuring canopy interception and forest floor evaporation in a beech (*Fagus Sylvatica* L.) forest in Luxembourg resolved this problem by stacking and weighing two aluminum basins with strain gauges, so that the evaporation rate of the precipitation interpreted by the forest floor could be measured when the event ceased [29, 30]. So, in the context of turfgrass patches, it may be possible to place a tipping-bucket pluviometer on top of a logging scale to track the rate of canopy evaporation during the storm and during the third stage of the process.

**Fig 4:**
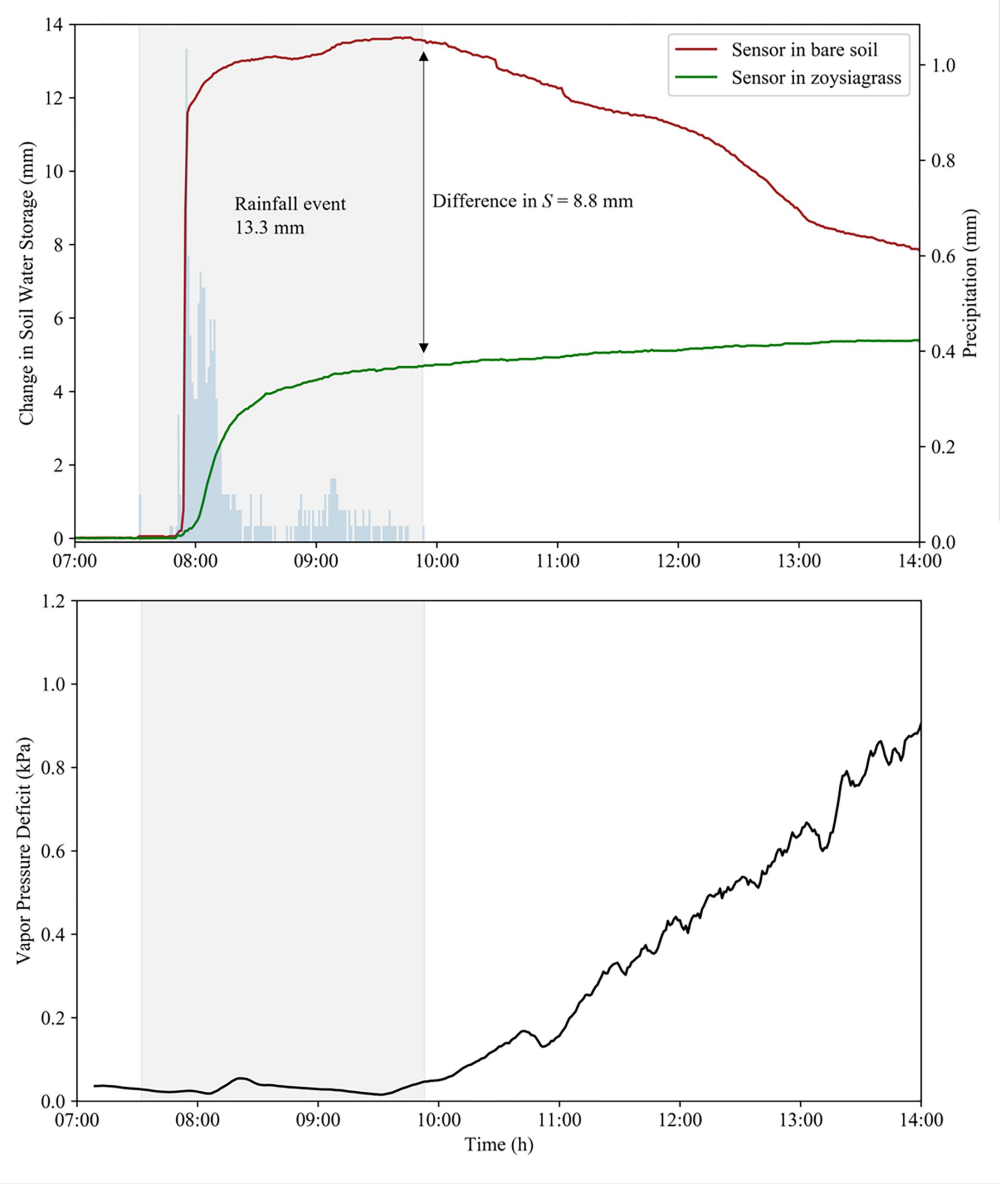
A) Changes in 1-minute soil water storage in the 0–12 cm soil layer measured with a vertically inserted soil water reflectometer in bare soil and in a Zoysiagrass canopy during a 13.3 mm rainfall event with a duration 2.5 hours on 22 May 2020 (Storm 14a); B) Vapor pressure deficit during and after the rainfall event. The post-storm decrease in soil water storage in Figure A is a result of soil moisture redistribution to deeper soil layer and evaporation driven by the increasing evaporative demand.

Furthermore, for a selected set of precipitation events, we investigated the impact of canopy interception by monitoring the change in soil water storage from 0–12 cm using soil moisture sensors. For instance, during a 13.3 mm rainfall event (storm 14a), soil water storage rapidly increased in a no canopy cover (Fig 4A). However, under the zoysiagrass canopy cover the increase in soil water storage was much slower and delayed. By the end of the rainfall event, soil water storage was 8.8 mm less under the zoysiagrass canopy than in the bare soil area. This further illustrates the impact that canopy interception has on near-surface soil moisture conditions and the soil water balance of shallow rooted plants, like turfgrass.

After the rainfall ceased, soil water storage decreased in the bare soil area with no canopy cover (Fig 4A), which was likely a result of multiple factors. First, the bare soil surface was directly exposed to solar radiation, which undoubtedly altered its energy balance and resulted in higher evaporation rates and faster drying rates than in the soils shaded by the zoysiagrass canopy [31, 32]. Increasing vapor pressure deficit after the rainfall also increased evaporation rates (Fig 4B), contributing to the faster drying (change in soil water storage) of bare soils compared with soils under the zoysiagrass. Finally, decreasing soil water storage in bare soils after the rainfall may have been caused in part by soil moisture redistribution to deeper soil layers (i.e., drainage).

Conversely, immediately after rainfall, the change in soil water storage was very slow in soils under the zoysiagrass canopy (Fig 4A). This was likely because evaporation in the zoysiagrass area was (initially) primarily from the wet canopy/thatch layer and not from the soil. This low evaporation from soils after rainfall is a positive aspect of having a turfgrass cover.

A better understanding of canopy interception can also be used to improve the estimation of other processes of the soil water balance, like runoff. For instance, in hydrology, the initial abstraction (*I_a_*) is a term used to describe precipitation storage prior to the beginning of water runoff. The *I_a_* accounts for the depression storage due to surface roughness, canopy and litter interception, and pre-ponding soil infiltration. A study in a non-infiltrating and highly compacted lawn found an *I_a_* (interception + depression storage) value of 6.8 mm, a value close to the observed value of 4.4 mm in our study [33]. Hence, measuring the amount of canopy interception may also be a key component to improve runoff prediction. To some extent, the meaning of *I_tf_* in the interception process can considered analogous to the meaning og *I_a_* in the runoff process.

**Fig 5:**
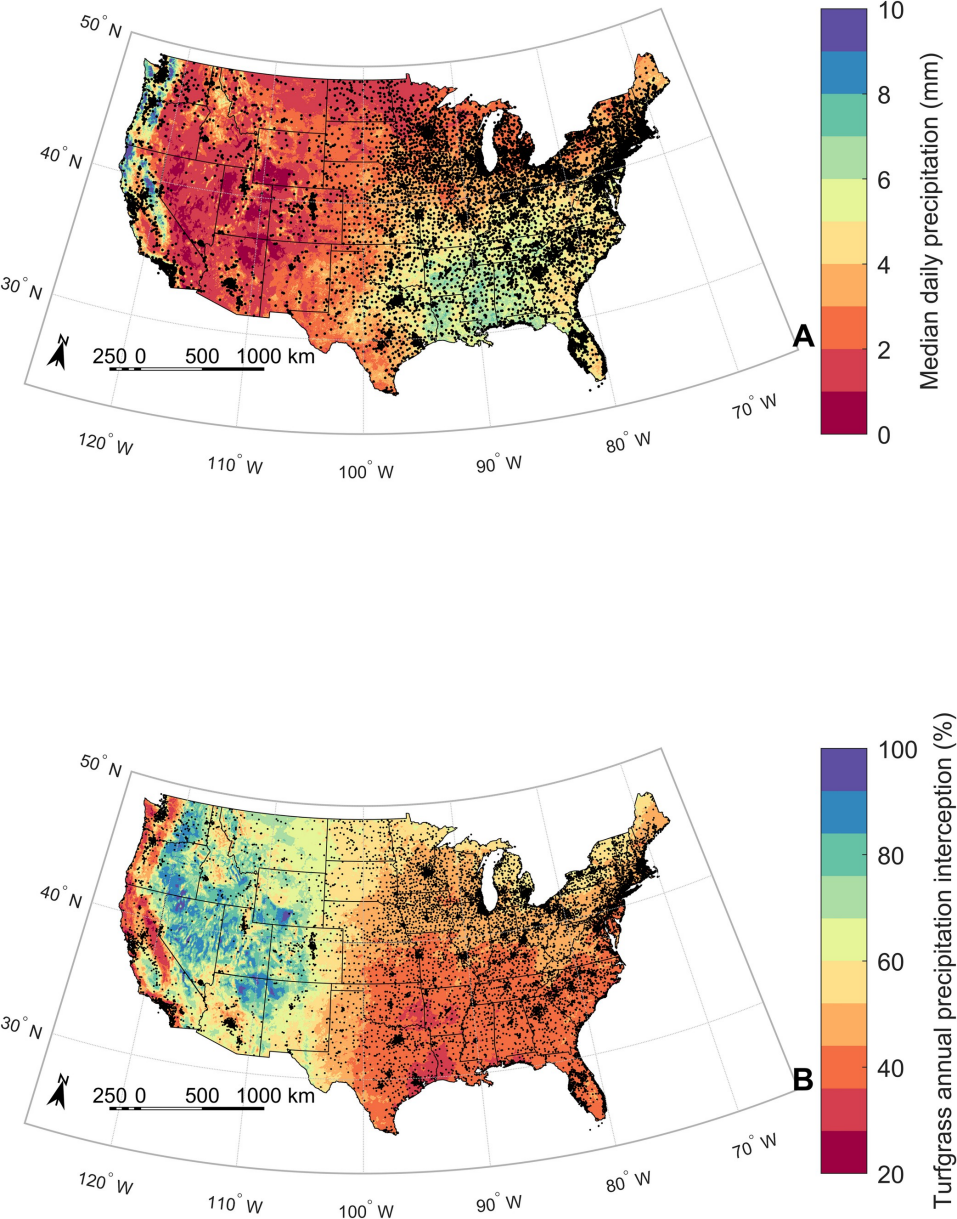
Maps showing the A) median daily precipitation totals for the contiguous United States in the period 2017–2020 from a multi-sensor gridded precipitation product at 4-km spatial resolution; and B) the estimated average percentage of annual precipitation intercepted by zoysiagrass and creeping bentgrass for the period 2017–2020 calculated using Eqs 2 and 3. Interception amount for daily precipitation events exceeding 45 mm was kept constant at a value of 11.4 mm [i.e., *I_tf_*+0.16(45−*I_tf_*), where *I_tf_* = 4.4 mm] since our study did not include larger events. Only days with precipitation >0 mm were used to compute the maps. Black markers represent the locations of golf courses throughout the U.S. Black markers represent the locations of golf courses throughout the U.S. Precipitation data was obtained from the U.S. National Weather Service (water.weather.gov/precip). The dataset of golf courses was obtained from the Golf Course Superintendents Association of America (GCSAA).

To illustrate the potential impact of precipitation interception by turfgrass canopies like zoysiagrass and bentgrass over a larger spatial extent, we also quantified the median daily precipitation amount and the potential canopy interception amount for the contiguous United States. For this analysis, we used a multi-sensor gridded precipitation product from the US National Weather Service at 4-km spatial resolution for the period of 1 January 2017 to 31 December 2020 (Fig 5). Interestingly, 72% of the area of the contiguous US has a median daily precipitation below the minimum interception storage capacity of 4.4 mm found in this study for zoysiagrass and bentgrass (Fig 5A). Considering the average interception losses during precipitation events >4.4 mm based on Eq 3, our analysis revealed that 45% of the area of the contiguous US could result in >50% of the annual precipitation being intercepted by canopies of zoysiagrass and bentgrass (Fig 5B). This exercise assumed that the findings in this study can be extrapolated to other regions. While these assumptions may not be valid over the entire territory and across seasons beyond those included in this study, this exercise allowed us to approximate the potential impact of turfgrass canopy interception on the national water balance.

One limitation of this study is that the turfgrass patches of zoysiagrass and bentgrass were clipped at a slightly (~3 mm difference) different height. This decision was intentional to match the typical heights in golf course fairways for each turfgrass. While the turfgrass height can affect the interception capacity of the patches, based on the storage capacity and patch thickness values reported in Table, this difference is likely in the order of a fraction of a millimeter of intercepted water. Similarly, the thickness of the thatch layer in our study is specific to the growth and management conditions in this study. Our study leaves several unanswered questions that warrant further research to better quantify the magnitude of canopy interpcetion and throughfall for different turfgrasses, clipping heights, and thatch thickness. A second limitation of this study is that the pluviometers were deployed 0.75 m above the ground (for practical reasons), which could have created a different aerodynamic resistance in the patches inside the pluviometer funnel compared to the surrounding grass. Thus, the evaporation rate of water droplets from the external surface of leaves and stems i) during rainfall events and ii) between rainfall events within the same storm. One possible modification to improve this technique and mitigate the issue of aerodynamic resistance is to bury the body of the pluviometers, so that the turfgrass patch inside the funnel is at the same level of the surrounding turfgrass. However, because of the relative humidity during rainfall events is typically ~99% it remains unclear whether the extra effort would result in measurable differences in canopy interception and throughfall.

## Conclusions

Our study consisted of quantifying throughfall and canopy interception of zoysiagrass and creeping bentgrass during rainfall events using a new method based on co-located pluviometers with and without a turfgrass patches. This new method enables simultaneous measurements of throughfall and canopy interception of turfgrass at high temporal resolution under natural rainfall conditions. The method of the co-located pluviometers allowed us to clearly identify well-defined stages of the interception process that may also apply to other land covers beyond turfgrass consisting of (1) complete precipitation interception by the canopy, (2) characterized by both throughfall and canopy interception, (3) and drying of the canopy after the precipitation has ceased.

Interception losses during the study period ranged from 34% in zoysiagrass to 47% in bentgrass. On average, the point of throughfall was 4.4 mm, suggesting that precipitation events <4.4 mm are unlikely to reach the soil surface in healthy turfgrass mowed at typical golf course fairway heights. Throughfall occurred when the turfgrass patches reached between 44 and 50% of their maximum water storage capacity. We encourage scientists and water managers to account for interception in water balance computations and irrigation scheduling routines. To our knowledge, this is the first study that provides detailed insights to understanding the interception dynamics in turfgrass and highlights the inefficient nature of small precipitation and irrigation events in turfgrass systems.

## Supporting information

S1 Data (XLSX)
